# Effects of Hydrochar Application on Soil Cadmium Immobilisation and Foxtail Millet Growth

**DOI:** 10.3390/toxics14100855

**Published:** 2026-09-25

**Authors:** Wenchi Guo, Yan Zhang, Jinpeng Yu, Yaping Li, Fei Han, Hong Pan, Hui Wang, Quangang Yang, Zhongchen Yang, Yanhong Lou, Yuping Zhuge

**Affiliations:** National Engineering Research Centre for Efficient Utilization of Soil and Fertilizer Resources, College of Resources and Environment, Shandong Agricultural University, Daizong Road, Tai’an 271018, China

**Keywords:** heavy metal, hydrochar, Cd immobilisation, bioavailability, foxtail millet

## Abstract

Cadmium (Cd) not only significantly threatens crop growth and food safety but also degrades soil quality and reduces microbial activity. Hydrochar is a carbon-rich material derived from biomass via hydrothermal carbonisation, due to its large specific surface area and abundant surface functional groups, shows great potential for immobilisation remediation of Cd-contaminated soils. We determined hydrochar’s (1) optimal application conditions for soil Cd immobilisation and (2) regulatory effects on foxtail millet growth and development, photosynthesis, antioxidant enzyme activity, resistance gene expression and Cd accumulation. Hydrochar was prepared at two temperatures (240 and 160 °C) and applied at two concentrations (1% and 2%). Hydrochar application reduced soil available Cd by 4.59–10.09% and shifted Cd fractions from acetic acid-extractable to residual forms. Soil organic matter (SOM) and microbial biomass carbon (MBC) increased. Hydrochar application increased plant antioxidant enzyme activity, downregulated *SiNRAMP5* and *SiHMA2* and upregulated *SiHMA3*, *SiPCS1* and *SiMT2*. Hydrochar prepared at 240 °C and applied at a 2% concentration exhibited the highest efficiency in immobilising Cd, enhancing plant photosynthesis and antioxidant defence, activating soil microorganisms and increasing SOM and MBC, offering a promising remediation strategy for weakly alkaline Cd-contaminated farmland in northern China.

## 1. Introduction

Heavy metal contamination of soil is a widely concerning environmental issue worldwide because of its serious threat to agricultural product safety and human health [1]. Cadmium (Cd) is one of the most hazardous elements causing farmland soil contamination in China, due to its high toxicity, concealment, mobility and bioaccumulation potential [2]. Cd contamination not only inhibits the normal growth and development of crops, reduces yield [3] and quality and tends to accumulate in edible parts, but also disrupts soil ecological functions by reducing soil organic matter (SOM) [4], suppressing enzyme activity and altering the microbial community composition [5]. Therefore, there is an urgent need for cost-effective remediation measures for Cd-contaminated soils. Remediation technologies for heavy metal-contaminated soils can be broadly categorised into three types: physical remediation, chemical remediation and bioremediation [6]. Among chemical remediation methods, in situ chemical immobilisation is characterised by simple operation [7], relatively low cost and compatibility with ongoing agricultural production, making it well suited to the arable land conditions in China and especially suitable for in situ remediation of farmlands with moderate-to-mild contamination [8]. The selection of appropriate immobilising materials is the key to the success of chemical remediation.

The most widely used in situ chemical immobilisation materials are clay minerals [9]; lime- [10], phosphate- and silicate-based materials [11]; and biochar. Biochar is one of the most extensively studied immobilisation materials in recent years, exhibiting superior adsorption and immobilisation capacity for heavy metals because of its large specific surface area and abundant surface functional groups [12]. Research shows that compared to cow manure alone, the application of cow manure-derived biochar is significantly more effective, reducing soil available Cd by 34.3–69.9% [13]. Furthermore, the remediation effects of biochar on Cd-contaminated soils vary with the pyrolysis temperature and application concentration [14]. Li et al. [15] observed that application of 5% biochar decreased the Cd concentration in pakchoi by 51% and increased the yield by 16%, outperforming the 1% and 2.5% concentrations. Nevertheless, biochar production typically requires high temperatures, resulting in considerable energy consumption. Moreover, as heavy metal stress decreases and soil microbial activity increases during soil remediation, the carbon source supply may become a critical limiting factor constraining the sustained effectiveness of remediation. However, for heavy metal-contaminated soils, remediation or safe use should address both the reduction in heavy metal availability and the improvement of soil quality.

In contrast, hydrochar is prepared via hydrothermal carbonisation at relatively low temperatures (180–250 °C), with lower energy consumption [16], eliminating the need for energy-intensive drying pretreatment. Consequently, its production cost and energy input are considerably lower than those of traditional biochar. Importantly, the mild hydrothermal carbonisation conditions enable maximum retention of oxygen-containing functional groups (e.g., hydroxyl and carboxyl groups) on the biomass surface, which efficiently immobilise heavy metals through complexation and chelation [17]. Furthermore, hydrochar application significantly increases SOM [18], providing an available carbon source for microorganisms. Hydrochar can also regulate resistance gene expression in plants [19], enhancing plants’ capacity for Cd sequestration and chelation detoxification. Furthermore, hydrochar modulates soil’s microbial community structure [20], reduces the abundance of heavy metal resistance genes in soil [21] and promotes functional microbial activity by improving soil’s physicochemical properties. However, the effects of hydrochar prepared at different temperatures and applied at different concentrations on plants and soil are unclear.

This study aimed to elucidate the immobilisation and remediation mechanisms of different hydrochar amendments in weakly alkaline Cd-contaminated soils and the remediation effects on plants. We hypothesised that hydrochar, prepared at different pyrolysis temperatures and applied at varying concentrations, could effectively reduce Cd bioavailability and alleviate Cd-induced stress in foxtail millet by modulating soil properties and microbial communities. To test these hypotheses, weakly alkaline Cd-contaminated soils were amended with hydrochar, and the overall soil remediation performance and plant physiological responses were systematically evaluated. The findings will provide a theoretical basis and technical support for the safe use of Cd-contaminated croplands in northern China.

## 2. Materials and Methods

### 2.1. Experimental Materials

Soil samples (0–20 cm depth) were collected from farmland in Tai’an City, Shandong Province, China. According to the Chinese Soil Taxonomy, the soil type was Typic Hapli-Udic Argosols (brown earth). The basic physicochemical properties were as follows: pH 7.97, SOM 20.58 g·kg^−1^, total nitrogen 1.46 g·kg^−1^, soil available phosphorus 23.61 mg·kg^−1^ and soil available potassium 82.51 mg·kg^−1^. Samples were air-dried and sieved through a 5 mm screen for subsequent pot trials. A Cd contamination concentration of 2.0 mg·kg^−1^ was set in this experiment. Soil samples were spiked with Cd^2+^ solution to a concentration of 2.0 mg·kg^−1^ (dry weight basis). A pre-prepared CdCl_2_·2.5H_2_O solution (obtained from Shanghai Macklin Biochemical Co., Ltd., Shanghai, China) (5.0 mg Cd·L^−1^, 2000 mL per pot) was thoroughly mixed with the sieved samples (5.0 kg) in plastic containers, with regular turning during the mixing process to ensure sufficient and uniform solution–soil contact. The mixed samples were aged under natural conditions for 45 d to allow the spiked Cd to equilibrate with the soil [22], yielding Cd-contaminated samples with a final Cd concentration of 2 mg·kg^−1^.

The foxtail millet cultivar used was Jigu 16, developed by the Shandong Academy of Agricultural Sciences, Jinan, China. Cornstalk-derived hydrochar was prepared by mixing cornstalk with deionised water at a 1:9 solid-to-liquid ratio, followed by hydrothermal carbonisation in a high-pressure autoclave at 160 and 240 °C for 2 h according to Yang et al. [23]. After carbonisation, the obtained hydrochar was washed repeatedly with deionised water, and then dried to a constant weight.

### 2.2. Experimental Design

The pot experiment was conducted under outdoor natural conditions at the Experimental Station of Shandong Agricultural University from June to October 2025. Foxtail millet seeds were sown in polyethylene pots (top diameter 21 cm, bottom diameter 15 cm, height 20 cm), each pot containing 5 kg of soil. Five experimental treatments were designed: CK, control; D1, 1% hydrochar produced at 160 °C (low temperature); D2, 2% hydrochar produced at 160 °C; G1, 1% hydrochar produced at 240 °C (high temperature); and G2, 2% hydrochar produced at 240 °C. The pot experiment was conducted during the growing season, and the same volume of water was applied to each pot throughout. Basal nutrients were added to the soil at the following rates (mg kg^−1^ soil): 118 mg of CO(NH_2_)_2_, 87 mg of (NH_4_)_2_HPO_4_ and 133 mg of K_2_SO_4_; 65 mg of CO(NH_2_)_2_ was top-dressed at the jointing stage (all fertilizers used were obtained from Shandong Nongda Fertilizer Sci. & Tech. Co., Ltd. Shandong, China), and each treatment included three biological replicates. Photosynthetic parameters of foxtail millet leaves were measured on day 30 of cultivation. Plant samples were collected on day 117 to determine physiological indicators. At harvest, both plant and soil samples were collected to analyse Cd concentrations in soil and plant tissues, as well as soil enzyme activity, soil organic matter (SOM), microbial biomass carbon (MBC), microbial community and other related parameters.

### 2.3. Measurements and Indicators

The pot experiment was conducted from 30 June to 25 October 2025. After the plants matured, soil and plant samples were collected. Rhizosphere soil was collected by gently shaking the roots to remove loosely adhering soil, and soil firmly attached to the root surface was collected with a sterile brush, for the determination of soil enzyme activity and microbial activity. Then, soil from each pot was poured out and thoroughly mixed for sampling to determine soil chemical properties. Plant samples were thoroughly washed with tap water to remove adhered soil particles, followed by rinsing thrice with deionised water, and then blotted dry with filter paper. Fresh soil and plant samples were stored in a freezer at −80 °C, while the remaining soil samples were naturally air-dried. The plants were separated into roots, stems, leaves and grains, which were then dried at 105 °C for 30 min, followed by drying at 80 °C to constant weight.

#### 2.3.1. Cd Concentration in Soil and Plants

Cd fractions were determined using the sequential extraction protocol established by the Community Bureau of Reference (BCR) [24]. The acid-extractable fraction was extracted with 0.11 mol·L^−1^ acetic acid, and the reducible fraction with 0.1 mol·L^−1^ hydroxylamine hydrochloride; the oxidisable fraction was first digested with 8.8 mol·L^−1^ hydrogen peroxide (pH = 2) and then extracted with 1 mol·L^−1^ ammonium acetate; and the residual fraction was digested with hydrochloric acid, nitric acid, hydrofluoric acid, and perchloric acid. Acetic acid, hydroxylamine hydrochloride, hydrogen peroxide, ammonium acetate, hydrochloric acid, nitric acid, hydrofluoric acid, and perchloric acid were obtained from Shanghai Macklin Biochemical Co., Ltd. Shanghai, China. Soil available Cd was measured using diethylenetriaminepentaacetic acid (DTPA) (obtained from Shanghai Macklin Biochemical Co., Ltd. Shanghai, China). Cd^2+^ concentration was measured using inductively coupled plasma–optical emission spectroscopy (ICP-OES; Thermo Scientific iCAP 7200; Thermo Fisher Scientific, Waltham, MA, USA); and Cd concentrations in various plant parts were determined using a nitric acid–perchloric acid mixture (9:1) and ICP-OES. All soil and plant samples were subjected to quality control using blank reagents, the certified reference material for soil (GBW07427, GSS-13), and the certified reference material for plants (GBW10036), and the recoveries were 98.3% and 97.7%, respectively. The bioconcentration factor (BCF) was calculated as the ratio of the Cd concentration in roots to that in soil, and the translocation factor (TF) was calculated as the ratio of the average Cd concentration in stems and leaves to that in roots [25].

#### 2.3.2. Soil Enzyme Activity

The activities of soil catalase (CAT), sucrase (SUC), urease (URE) and alkaline phosphatase (ALP) were determined using a double-antibody sandwich enzyme-linked immunosorbent assay. Absorbance was measured at 450 nm, and enzyme activity concentrations were calculated from standard curves [26].

#### 2.3.3. Plant Growth Characteristic

Plant height and stem diameter were measured using a vernier calliper, and plant weights were recorded using an analytical balance with a precision of 0.0001 g. Leaf area was calculated as leaf length × maximum width × 0.75.

#### 2.3.4. Leaf Biochemical Parameters and Root Resistance Genes

Proline, H_2_O_2_, malondialdehyde (MDA) and superoxide radical (O_2_^−^) contents and O_2_^−^ production rate were determined using ADS-W-AJS004, ADS-W-YH001, ADS-W-YH002 and ADS-W-YH008 assay kits (Jiangsu Aidisheng Biological Technology Co., Ltd., Yancheng, China). Superoxide dismutase (SOD), peroxidase (POD), CAT and glutathione reductase (GR) activities were determined using F5056-A, F5058-A, F4957-A, and F5015-A assay kits (Jiangsu Aidisheng Biological Technology Co., Ltd.), respectively.

The relative expression levels of five genes encoding *SiNRAMP5*, *SiHMA2*, *SiHMA3*, *SiPCS1* and *SiMT2* in foxtail millet leaves were determined. Briefly, total RNA was extracted using the FastPure Plant Total RNA Isolation Kit (RC401; Vazyme, Nanjing, China). After quality assessment with a Nanodrop 2000 (Thermo Fisher Scientific), RNA was reverse-transcribed into complementary DNA (cDNA) using the HiScript^®^ II 1st Strand cDNA Synthesis Kit (R211-01; Vazyme). Quantitative reverse transcription polymerase chain reaction (qRT-PCR) was performed using the 2× ChamQ SYBR Colour qPCR Master Mix (Q441-02; Vazyme), and relative gene expression was calculated using the 2^−ΔΔCt^ method [27,28].

#### 2.3.5. Leaf Photosynthesis and Gas Exchange

The following photosynthetic parameters of foxtail millet were analysed using a portable photosynthesis system (LI-6800, LI-COR Inc., Lincoln, NE, USA) from 9:00 a.m. to 10:00 a.m. with a light intensity of 1000 μmol m^−2^ s^−1^ at a leaf temperature of 25 °C in a leaf chamber supplied with a 300 μmol mol^−1^ CO_2_ concentration: intercellular CO_2_ concentration (Ci), net photosynthetic rate (Pn), stomatal conductance (Gs), transpiration rate (Tr) and maximum quantum efficiency of Photosystem II (PSII; Fv/Fm).

#### 2.3.6. Soil Microbial Properties and SOM

Total soil microbial DNA was extracted and subjected to metagenomic sequencing on the NovaSeq platform (Illumina, San Diego, CA, USA). Raw sequencing data were quality-filtered, followed by sequence assembly, gene prediction and construction of a non-redundant gene catalogue. Microbial taxonomic annotation was performed based on the National Center for Biotechnology Information’s Non-Redundant Protein Database.

To determine soil MBC content, according to GB/T 39228-2020, fresh soil was extracted with 0.5 mol L^−1^ of K_2_SO_4_ (obtained from Shanghai Macklin Biochemical Co., Ltd. Shanghai, China) solution after chloroform fumigation, and the organic carbon concentration in the extract was measured using a total organic carbon analyser (Shimadzu TOC-500; Shimadzu Corporation, Kyoto, Japan). SOM content was measured using the K_2_Cr_2_O_7_ external heating method. The soil microbial quotient (SMQ) was then calculated as the ratio of MBC to SOM.

#### 2.3.7. Characterisation of Hydrochar

Hydrochar’s specific surface area and pore structure were determined using a fully automatic surface area and porosity analyser (ASAP 2460; Micromeritics, Norcross, GA, USA) [29]. Hydrochar’s surface morphology was observed using scanning electron microscopy (SEM; Tescan MIRA LMS; Tescan, Czech Republic). Fourier transform infrared (FTIR) spectra were obtained using a Nicolet iS20 spectrometer (Thermo Fisher Scientific) via the KBr pellet method in the 400–4000 cm^−1^ range at a 4 cm^−1^ resolution.

### 2.4. Statistical Analysis

Microsoft Excel 2019 (Microsoft Corporation, Redmond, WA, USA) and SPSS version 21 (IBM Corp., Armonk, NY, USA) were used for data processing. The significance of differences among all treatments was determined using one-way analysis of variance (ANOVA). The main and interactive effects of hydrochar preparation temperature and concentration on all measured parameters were evaluated using two-way ANOVA. Duncan’s multiple-range test was performed for post hoc comparisons (*p* = 0.05). Graphs were compiled using Origin 2024 (OriginLab, Northampton, MA, USA).

## 3. Results

### 3.1. Cd Concentration in Soil

Compared with CK, hydrochar treatments decreased soil available Cd by 4.59–10.09%. Soil available Cd significantly decreased by 3.77% with G1 compared to D1 and by 5.58% with G2 compared to D2. No significant differences were observed due to different hydrochar concentrations under the same temperature condition. ANOVA showed that hydrochar preparation temperature significantly affected soil available Cd concentration (*p* < 0.001), while hydrochar concentration and its interaction with preparation temperature showed no significant effects (Figure 1a).

Compared with CK, hydrochar application decreased acid-extractable Cd by 6.42–23.18% but increased oxidisable Cd and residual Cd by 2.39–10.60% and 4.08–12.89%, respectively; the reducible fraction remained statistically unchanged. G1 and G2 exhibited superior Cd immobilisation performance to D1 and D2, respectively. Furthermore, 2% hydrochar was more effective than 1% hydrochar in Cd immobilisation (Figure 1b).

### 3.2. Cd Concentration in Plants

Compared with CK, hydrochar application significantly reduced Cd concentrations in all tested plant parts: by 11.67–33.56% in roots, by 21.72–46.23% in stems, by 21.62–48.81% in leaves and by 4.20–22.53% in grains.

The Cd concentration in roots, stems, leaves and grains significantly decreased by 13.88%, 14.13%, 22.55% and 11.75%, respectively, with G1 compared to D1; by 21.38%, 26.17%, 31.78% and 13.05%, respectively, with G2 compared to D2; by 4.32%, 6.80%, 4.25% and 6.99%, respectively, with D2 compared to D1; and by 12.65%, 19.86%, 15.68% and 8.38%(Figure 2a), respectively, with G2 compared to G1. ANOVA showed that hydrochar preparation temperature, concentration and their interaction all highly significantly affected root, stem and leaf Cd concentrations (all *p* < 0.001). However, only temperature and concentration significantly affected the grain Cd concentration (*p* < 0.001); their interaction was not significant (Table S1).

The BCF and TF showed decreasing trends across treatments in the order CK > D1 > D2 > G1 > G2. The BCF and TF decreased by 10.16% and 4.94%, respectively, with G1 compared to D1; by 16.74% and 9.54%, respectively, with G2 compared to D2; by 4.15% and 1.32%, respectively, with D2 compared to D1; and by 11.17% and 6.09%, respectively, with G2 compared to G1 (Figure 2b).

### 3.3. Soil Enzyme Activities

Compared with D1, G1 significantly increased soil URE, SUC, CAT and ALP activities by 9.47%, 19.70%, 7.85% and 10.38%, respectively. Compared with D2, G2 significantly increased URE, SUC and CAT activities by 14.20%, 24.83% and 16.65%, respectively; ALP activity was not significantly different.

Furthermore, compared with D1, D2 significantly increased ALP activity by 11.03% and significantly decreased SUC activity by 3.16%; URE and CAT activities were not significantly different. Compared with G1, G2 significantly increased URE, SUC and CAT activities by 5.48%, 1.00% and 8.20%, respectively; ALP activity was not significantly different (Figure 3).

ANOVA showed that hydrochar preparation temperature highly significantly affected URE, SUC, CAT and ALP activities (all *p* < 0.001). Hydrochar concentration significantly affected URE and SUC activities (both *p* < 0.05) and CAT and ALP activities (both *p* < 0.001). The temperature–concentration interaction highly significantly affected SUC and CAT activities (both *p* < 0.001) but not URE or ALP activity (Table S1).

### 3.4. Plant Growth

Compared with D1, G1 significantly increased plant height, stem diameter, leaf area and aboveground biomass by 18.86%, 33.33%, 29.10% and 24.51%, respectively, while belowground biomass was not significantly different. Compared with D2, G2 significantly increased plant height, stem diameter, leaf area, aboveground biomass and belowground biomass by 27.69%, 30.04%, 84.20%, 56.27% and 123.78%, respectively.

Furthermore, compared with D1, D2 significantly increased stem diameter by 42.93% and significantly decreased belowground biomass by 32.10%; plant height, leaf area and aboveground biomass were not significantly different. Compared with G1, G2 significantly increased plant height, stem diameter and leaf area by 14.60%, 39.39% and 42.23%, respectively, while aboveground and belowground biomass were not significantly different (Table 1).

### 3.5. Plant Antioxidant System

Compared with CK, hydrochar treatments significantly increased GR, SOD, CAT and POD activities by 29.62–59.99%, 32.21–45.09%, 27.87–50.91% and 18.83–66.60%, respectively. GR, SOD, CAT and POD activities significantly increased by 19.32%, 5.73%, 12.62% and 25.00%, respectively, with G1 compared to D1; by 13.40%, 5.91%, 8.53% and 23.34%, respectively, with G2 compared to D2; by 8.85%, 3.61%, 8.74% and 13.67%, respectively, with D2 compared to D1; and by 3.44%, 3.79%, 4.79% and 12.16%, respectively, with G2 compared to G1 (Figure 4).

ANOVA showed that both hydrochar preparation temperature and hydrochar concentration highly significantly affected GR, SOD, POD and CAT activities (all *p* < 0.001). The temperature–concentration interaction significantly affected GR activity (*p* < 0.01) but not SOD, POD or CAT activity (Table S1).

### 3.6. Photosynthetic Parameters

Compared with CK, D1 and D2 significantly decreased Ci by 4.39% and 13.70%, respectively, while G1 and G2 significantly increased it by 25.40% and 37.15%, respectively. Furthermore, G1 and G2 significantly increased Pn by 30.67% and 44.44%, respectively, while D1 and D2 showed no significant change. Moreover, D1 and D2 significantly decreased Gs by 17.19% and 13.65%, respectively, while G1 and G2 significantly increased it by 14.55% and 27.11%, respectively. In addition, D2, G1 and G2 significantly increased Tr by 20.88%, 21.84% and 61.05%, respectively, while D1 showed no significant change. Finally, D1 and D2 significantly decreased Fv/Fm by 0.92% and 1.14%, respectively, while G1 and G2 significantly increased it by 1.89% and 2.68%, respectively.

Furthermore, Ci, Pn, Gs, Tr and Fv/Fm significantly increased by 31.16%, 36.02%, 38.34%, 19.25% and 2.84%, respectively, with G1 compared to D1 and by 58.93%, 50.56%, 47.19%, 33.23% and 3.87%, respectively, with G2 compared to D2.

Moreover, compared with D1, D2 significantly decreased Ci by 9.74%; significantly increased Gs and Tr by 4.29% and 18.31%, respectively; and showed no significant difference in Pn and Fv/Fm. Similarly, compared with G1, G2 significantly increased Ci, Pn, Gs, Tr and Fv/Fm by 9.36%, 10.54%, 10.96%, 32.18% and 0.78%, respectively (Figure 5).

ANOVA showed that hydrochar preparation temperature highly significantly affected Tr, Gs, Pn, Ci and Fv/Fm (all *p* < 0.001). Hydrochar concentration highly significantly affected Tr and Gs (both *p* < 0.001) and Pn (*p* < 0.01) but showed no significant effects on Ci or Fv/Fm. The temperature–concentration interaction highly significantly affected Tr and Ci (both *p* < 0.001), Gs and Pn (both *p* < 0.01) and Fv/Fm (*p* < 0.05) (Table S1).

### 3.7. Plant Physiological and Biochemical Indicators

Compared with CK, D2, G1 and G2 significantly decreased O_2_^−^ content by 3.01%, 2.60% and 17.27%, respectively, while D1 showed no significant difference. All hydrochar treatments significantly decreased O_2_^−^ production rate (25.49–37.46%). D1 and D2 significantly increased MDA content by 34.15% and 17.73%, respectively, while G1 and G2 significantly decreased it by 15.47% and 28.65%, respectively. D2, G1 and G2 significantly decreased H_2_O_2_ content by 16.72%, 14.68% and 33.68%, respectively, while D1 showed no significant change. All hydrochar treatments significantly decreased PRO content (12.81–52.27%).

Furthermore, O_2_^−^ content, O_2_^−^ production rate, MDA, H_2_O_2_ and PRO significantly decreased by 2.50%, 6.52%, 36.99%, 11.32% and 31.89%, respectively, with G1 than compared to D1 and by 14.68%, 7.77%, 39.39%, 20.35% and 32.66%, respectively, with G2 compared to D2. Moreover, O_2_^−^ content, O_2_^−^ production rate, MDA, H_2_O_2_ and PRO significantly decreased by 2.91%, 8.99%, 12.24%, 13.47% and 18.69%, respectively, with D2 compared to D1 and by 15.05%, 10.20%, 15.59%, 22.24% and 19.61%, respectively, with G2 compared to G1 (Figure 6).

ANOVA showed that hydrochar preparation temperature highly significantly affected O_2_^−^ content, O_2_^−^ production, MDA, H_2_O_2_ and PRO (all *p* < 0.001). Hydrochar concentration highly significantly affected O_2_^−^ content, O_2_^−^ production, MDA and H_2_O_2_ (all *p* < 0.001), as well as PRO (*p* < 0.01). The temperature–concentration interaction highly significantly affected only O_2_^−^ content (*p* < 0.001) but not O_2_^−^ production, MDA, H_2_O_2_ or PRO (Table S1).

### 3.8. Resistance Genes in Plants

Compared with CK, hydrochar application significantly decreased *SiNRAMP5* and *SiHMA2* expression by 14.35–64.43% and 19.06–44.12%, respectively. *SiNRAMP5* and *SiHMA2* expression significantly decreased by 48.74% and 21.25%, respectively, with G1 compared to D1; by 51.68% and 25.90%, respectively, with G2 compared to D2; by 14.07% and 6.84%, respectively, with D2 compared to D1; and by 19.01% and 12.33, respectively, with G2 compared to G1.

Compared with CK, hydrochar application significantly increased *SiHMA3*, *SiPCS1* and *SiMT2* expression by 51.42–105.43%, 98.62–179.99% and 43.17–125.55%, respectively. Compared with D1, G1 significantly increased *SiHMA3* and *SiMT2* expression by 32.47% and 33.08%, respectively, while *SiPCS1* expression showed no significant difference. Compared with D2, G2 significantly increased *SiHMA3*, *SiPCS1* and *SiMT2* expression by 51.07%, 40.39% and 53.32%, respectively.

Moreover, compared with D1, D2 significantly decreased *SiHMA3* expression by 10.20%, while *SiPCS1* and *SiMT2* expression showed no significant difference. Finally, compared with G1, G2 significantly increased *SiPCS1* and *SiMT2* expression by 22.11% and 18.38%, respectively, while *SiHMA3* expression showed no significant difference (Figure 7).

ANOVA showed that hydrochar preparation temperature highly significantly affected *SiNRAMP5*, *SiHMA2*, *SiHMA3*, *SiPCS1* and *SiMT2* expression (all *p* < 0.001). Hydrochar concentration significantly affected *SiNRAMP5*, *SiHMA2*, *SiPCS1* and *SiMT2* expression (all *p* < 0.001) but not *SiHMA3* expression. The temperature–concentration interaction highly significantly affected *SiPCS1* and *SiMT2* (both *p* < 0.001) and *SiHMA3* (*p* < 0.05) expression but not *SiNRAMP5* or *SiHMA2* expression (Table S1).

### 3.9. Soil Microbial Community, MBC and SOM

At the phylum level, hydrochar application significantly altered the composition of soil bacterial communities. Compared with CK, all hydrochar treatments significantly increased the relative abundance of *Bacillota* (178–290%), G1 and G2 significantly decreased the relative abundance of *Actinomycetota* (23–25%), and D2 and G2 significantly enriched the relative abundance of *Pseudomonadota* (17–18%; *p* < 0.05). Notably, the relative abundance of *Nitrososphaerota* showed a temperature-dependent response: it significantly increased by 29.3% with G1 but significantly decreased by 28.4% with D2 (*p* < 0.05), indicating a clear temperature–concentration interaction. Furthermore, the uncultured phyla *Candidatus_Binatota* exhibited a dose-dependent increase under high-temperature treatments, with its abundance being 68.1% higher with G2 compared with CK, indicating a potential ecological role in heavy metal remediation (Figure 8a).

Compared with CK, hydrochar application significantly increased SOM, MBC and SMQ by 28.74–63.39%, 34.60–107.39% and 4.56–26.93%, respectively. SOM, MBC and SMQ significantly increased by 8.01%, 22.13% and 13.08%, respectively, with G1 compared to D1; by 15.36%, 31.58% and 14.06%, respectively, with G2 compared to D2; by 10.02%, 17.08% and 6.42%, respectively, with D2 compared to D1; and by 17.51%, 26.14% and 7.35%, respectively, with G2 compared to G1 (Figure 8b).

### 3.10. SEM, BET and FTIR Analyses of Hydrochar

The specific surface and mesopore areas of hydrochar-240 °C significantly increased by 274.70% and 443.48%, respectively, compared to hydrochar-160 °C; the micropore area showed no significant change (Table 2). These results indicate that increasing the preparation temperature substantially promotes the development of mesoporous structures in hydrochar, thereby increasing its specific surface area. SEM images further confirmed this observation, showing that hydrochar-240 °C exhibited a rougher and more porous morphology, whereas hydrochar-160 °C displayed a relatively smooth, compact surface (Figure 9a,b). Furthermore, compared with hydrochar-160 °C, hydrochar-240 °C exhibited a more distinct carboxyl C=O stretching vibration peak at 1703 cm^−1^, indicating that a higher preparation temperature promotes carboxyl formation (Figure 9c).

### 3.11. Comprehensive Evaluation

A comprehensive evaluation based on correlation analysis (Figure 10) and two-way ANOVA results was conducted on the combined effects of hydrochar application on soil quality and plant physiological responses. Correlation analysis indicated that both hydrochar preparation temperature and application concentration were significantly correlated with most measured parameters. Specifically, temperature was negatively correlated with soil available Cd and Cd concentration in roots, stems, leaves and grains and positively correlated with soil enzyme activities (URE, SUC, CAT and ALP), photosynthetic parameters (Pn, Gs and Tr), antioxidant enzyme activities (SOD, POD, CAT and GR), SOM, MBC and resistance gene expression (*SiHMA3*, *SiPCS1* and *SiMT2*). Hydrochar concentration also showed positive correlations with most indicators, indicating that Cd accumulation inhibits soil biological activity and plant photosynthesis, while hydrochar application alleviates this stress.

Two-way ANOVA further showed that temperature highly significantly affected all measured indicators (*p* < 0.001), while the effects of concentration and temperature–concentration interaction varied among indicators. Temperature was the dominant factor driving the reduction in soil available Cd, enzyme activity enhancement, photosynthesis improvement and upregulation of resistance gene expression. The 2% concentration generally outperformed the 1% concentration across most indicators.

In summary, hydrochar application can synergistically improve soil–plant system health through the following pathways: reducing soil available Cd concentration and inhibiting Cd translocation to aboveground plant parts (stems, leaves and grains) via *SiNRAMP5* and *SiHMA2* downregulation and *SiHMA3*, *SiPCS1* and *SiMT2* upregulation; improving soil quality by significantly increasing SOM, MBC and activities of URE, SUC, CAT and ALP; enhancing plant physiological tolerance by increasing SOD, POD, CAT and GR activities and PRO content and decreasing MDA and H_2_O_2_ contents; and improving photosynthetic performance by increasing Pn, Gs, Tr and Fv/Fm and decreasing Ci. Considering all parameters, G2 treatment exhibited the best overall performance, effectively promoting soil microbial activity and SOM enhancement, strengthening the plant antioxidant defence system and reducing Cd bioavailability and its accumulation in grains. This treatment shows promising potential for remediation of heavy metal-contaminated farmland.

## 4. Discussion

The immobilisation efficiency of Cd is affected by material preparation [30]. For example, compared to sewage sludge biochar prepared at 350 °C, that prepared at 450 °C reduces the Cd mobile fraction in soil by 10.2% [31]. This phenomenon is attributed to the increased surface area and enhanced functional groups of the immobilising materials at higher preparation temperatures [32]. The specific surface area of biochar significantly increases with increasing pyrolysis temperature within the range of 400–700 °C [33], and hydrochar prepared at higher temperatures preserves a greater abundance of oxygen-containing functional groups [34]. In this study, the hydrochar preparation temperature had a highly significant effect on Cd immobilisation and plant performance, with high-temperature (240 °C) hydrochar outperforming its low-temperature (160 °C) counterpart. Compared with hydrochar-160 °C, hydrochar-240 °C exhibited significantly increased the specific surface and mesopore areas, and a carboxyl C=O peak appeared in its FTIR spectrum; this characteristic peak was absent in hydrochar-160 °C. In addition, hydrochar-240 °C showed superior Cd immobilisation efficiency, reduced soil available Cd and promoted the transformation of Cd from the acetic acid-extractable fraction to the residual fraction, thereby reducing its mobility. These findings are consistent with those of Zong et al. [35], who reported that compared with 300 °C biochar, 500 °C biochar reduced DTPA-extractable Cd by 39.7% and increased the residual Cd fraction from ~20% to >40%.

After Cd immobilisation, its toxicity significantly reduces, while its plant uptake and translocation decrease. Consequently, the stress on plants is alleviated, thereby promoting plant growth [36]. This study showed that hydrochar application effectively promoted plant photosynthesis, with the G2 treatment showing the best photosynthetic performance, as indicated by a 2.68% increase in Fv/Fm. Further analysis revealed that compared with hydrochar concentration, hydrochar preparation temperature exerted a significantly stronger regulatory effect on photosynthetic parameters and was thus identified as the dominant factor influencing photosynthetic efficiency. This is because the stronger Cd^2+^ immobilisation by hydrochar-240 °C reduced the Cd flux into chloroplasts, thereby protecting the PSII function [37]. This protective effect not only was manifested at the level of photosynthetic organs but also extended to the whole-plant antioxidant defence system and the rhizosphere soil’s micro-ecological environment. Compared with hydrochar-160 °C, hydrochar-240 °C significantly increased the activities of plant antioxidant enzymes (SOD, POD and CAT), indicating more effective alleviation of Cd-induced oxidative damage. Similarly, after hydrothermal treatment, biochar significantly increases SOD, POD and CAT activities in mung bean seedlings [38]. In this study, soil enzyme activities significantly increased in the high-temperature groups compared to the low-temperature treatment groups. This is consistent with previous findings on hydrochar derived from aloe vera residue [39], indicating that hydrochar can stimulate microbial activity by providing labile organic carbon or altering soil’s physicochemical properties, thereby enhancing soil enzyme activities. Notably, the hydrochar-160 °C exhibited some distinct effects, including a decrease in SUC activity by 4.6–6.8% and an increase in MDA content by 17.7–34.1%. This may be attributed to the retention of more organic acids or soluble phenolic compounds in hydrochar-160 °C [40], which exerts short-term stress on soil microorganisms and plants.

The optimal concentration differs among immobilisation materials and also varies depending on the degree of soil contamination. Ding et al. [41] reported that vermiculite-modified biochar Cd availability decreased by 23.73–31.29% as the biochar concentration increased from 0.1% to 3%. Vuong et al. [42] reported that 10% taro stem biochar addition achieved a 20.6% higher immobilisation rate of exchangeable Pb than the 6% addition. Studies have also shown that Cd immobilisation efficiency at a >5% concentration is lower than that at a 2–3% concentration [43]. In this study, the 2% concentration outperformed the 1% concentration in terms of plant uptake and translocation of Cd, and activities of soil enzymes and plant antioxidant enzymes. The effect of concentration was significant for most parameters but with lower intensity than that of temperature. Similarly, Khadem and Raiesi [44] found that the positive effects of biochar addition on soil enzymes were much greater at a 1% than at a 0.5% concentration for all assayed enzymes. Ay et al. [45] found that compared with 1% biochar, 2% biochar increased CAT activity by 93.3% and SOD activity by 41.9% under Cu stress, which is consistent with the dose-dependent effects reported earlier [46]. Although hydrochar, as a novel and effective immobilisation material, significantly reduces Cd stress on plants and activates soil microorganisms, its effectiveness in reducing soil available Cd is limited. Therefore, further in-depth studies are needed to enhance hydrochar’s Cd immobilisation efficiency by exploring alternative preparation temperatures, concentrations and/or modification strategies. In addition, elucidating hydrochar’s intrinsic mechanisms of action is crucial for a comprehensive understanding of its application value in mitigating heavy metal toxicity in plants.

## 5. Conclusions

Hydrochar application represents an effective strategy for decreasing soil cadmium bioavailability, mitigating cadmium stress on plants and enhancing soil quality. Notably, temperature is the key factor affecting soil cadmium immobilisation, plant growth and soil quality. At the same concentration, hydrochar produced at 240 °C (high temperature) performed better than hydrochar produced at 160 °C (low temperature); that is, G1 and G2 performed better than D1 and D2, respectively. Furthermore, under the same temperature condition, 2% hydrochar was more effective than 1% hydrochar; that is, D2 and G2 performed better than D1 and G1, respectively. G2 exhibited the best performance in decreasing soil cadmium bioavailability and facilitating cadmium transformation to the residual fraction, reducing plant cadmium uptake and translocation, promoting plant growth and, concurrently, improving soil quality and activating soil microorganisms. These results confirm our hypothesis and elucidated the immobilisation and remediation mechanisms of hydrochar in weakly alkaline cadmium-contaminated soils. However, cadmium immobilisation and plant growth were relatively limited, so future research should focus on enhancing the immobilisation performance and stability related to cadmium.

## Figures and Tables

**Figure 1:**
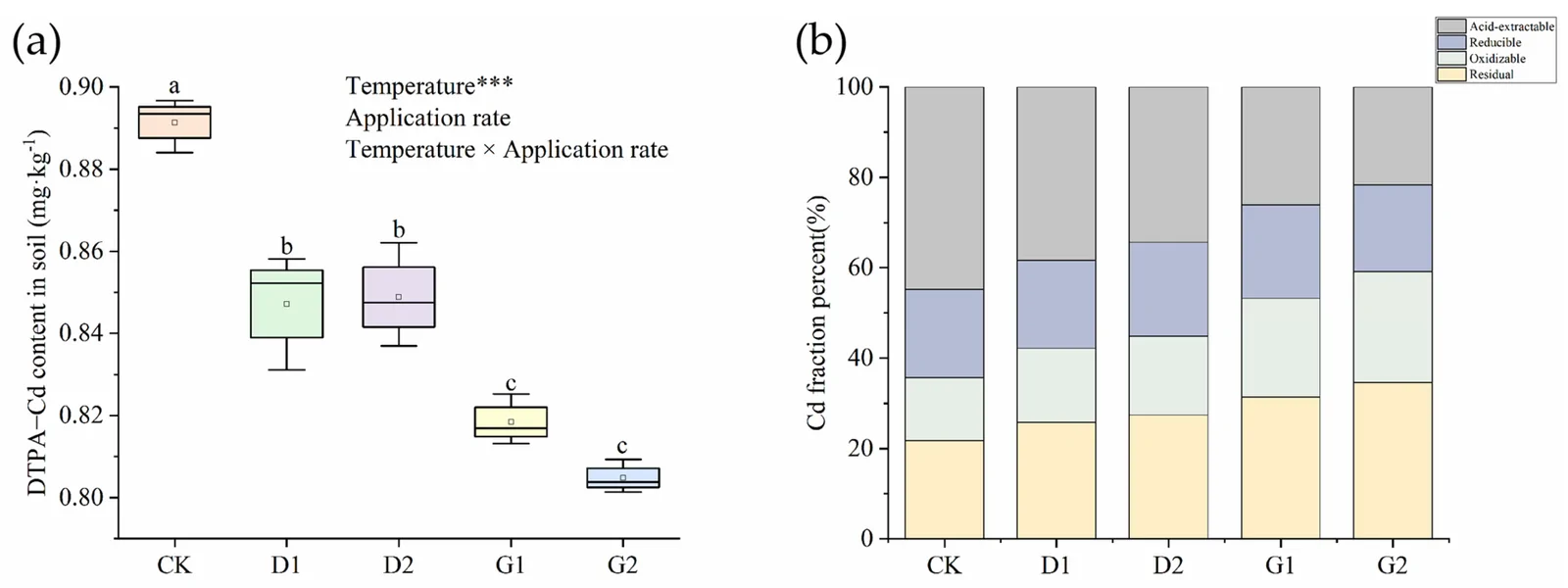
DTPA-extractable Cd concentration (**a**) and Cd fraction distribution (**b**) in soil under different hydrochar treatments. Treatments: CK, no hydrochar addition; D1, 1% hydrochar produced at 160 °C; D2, 2% hydrochar produced at 160 °C; G1, 1% hydrochar produced at 240 °C; and G2, 2% hydrochar produced at 240 °C. Different lowercase letters represent significant differences between different treatments at *p* < 0.05; *** represent significant at the 0.001 level (*p* < 0.001).

**Figure 2:**
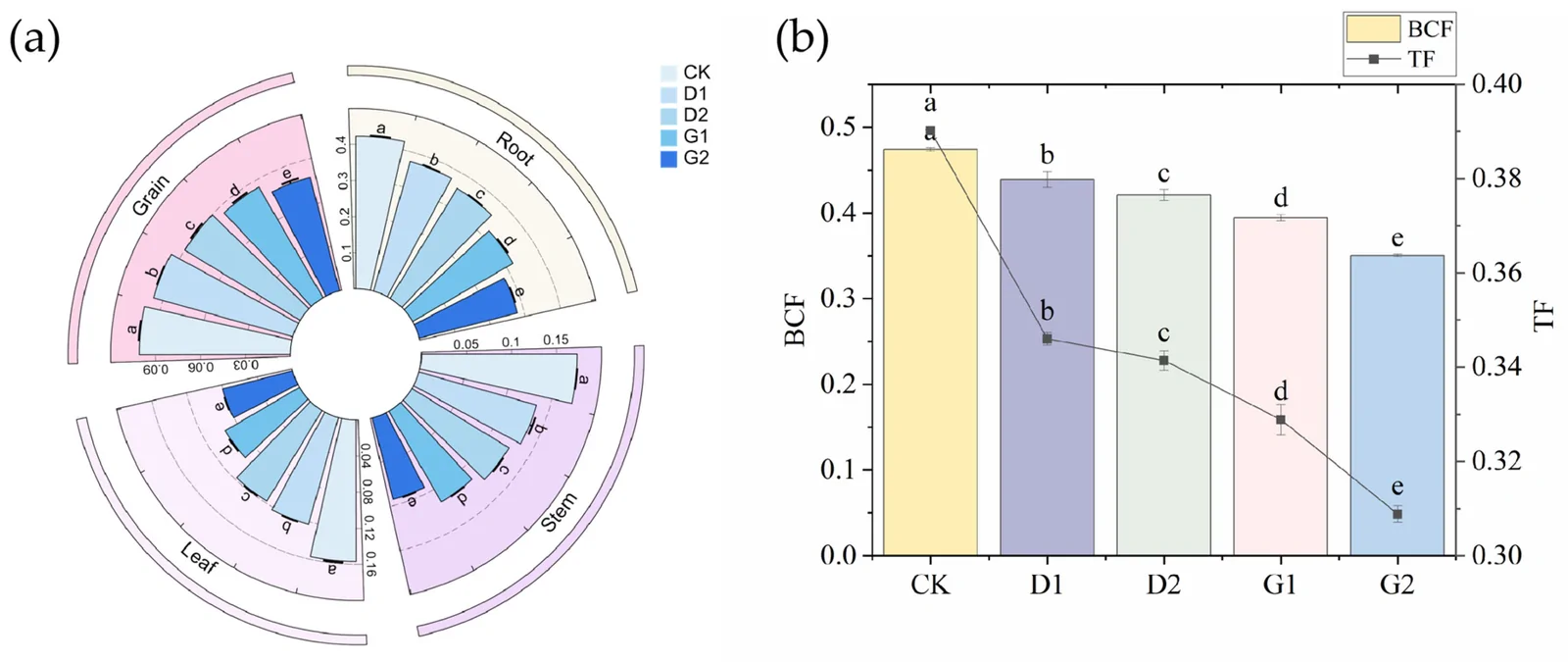
Cd concentrations in different plant organs (root, stem, leaf, and grain) (**a**), and the BCF and TF of Cd in foxtail millet under different treatments (**b**). Treatments: CK, no hydrochar addition; D1, 1% hydrochar produced at 160 °C; D2, 2% hydrochar produced at 160 °C; G1, 1% hydrochar produced at 240 °C; and G2, 2% hydrochar produced at 240 °C. Different lowercase letters represent significant differences between different treatments at *p* < 0.05.

**Figure 3:**
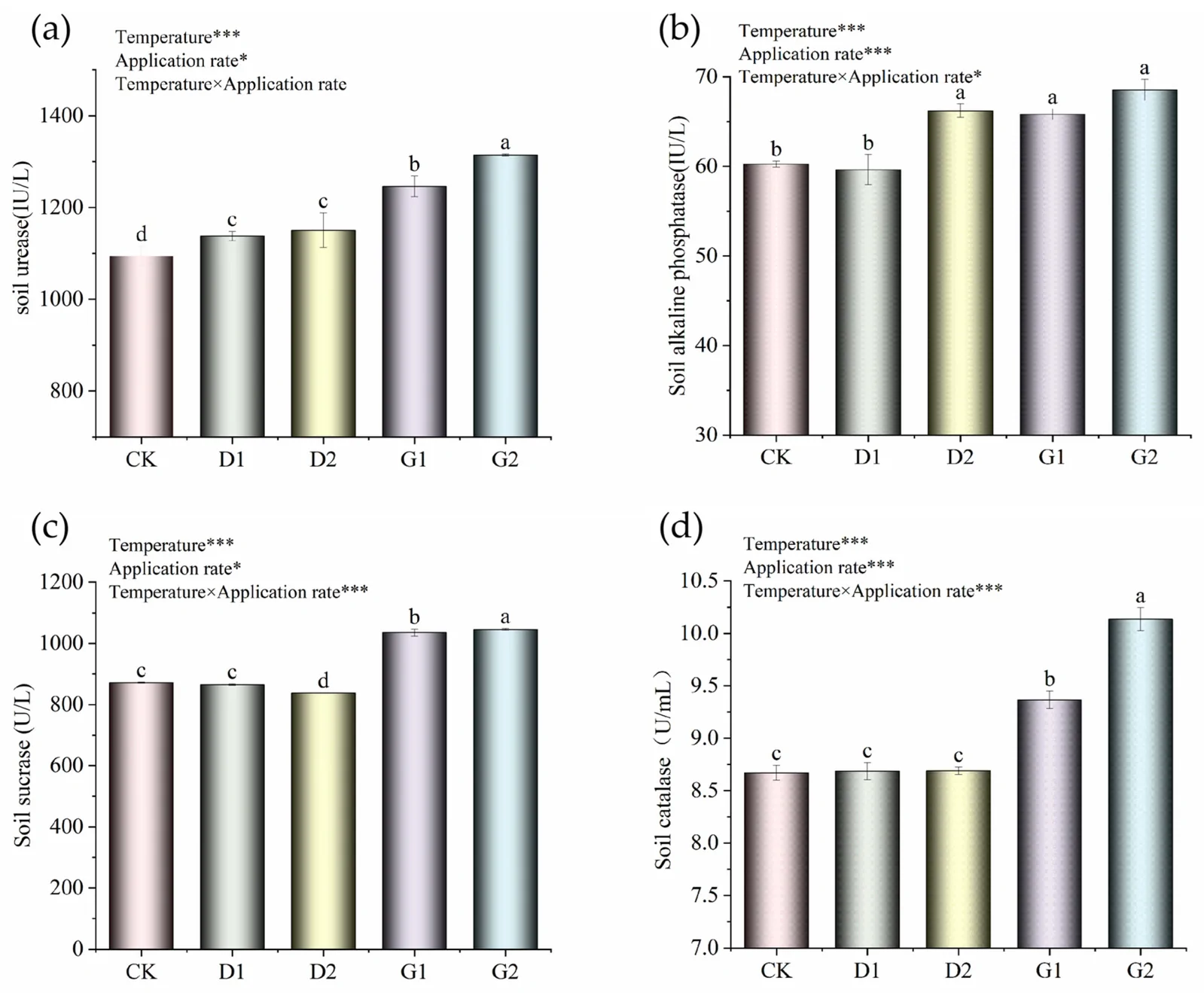
Activities of (**a**) soil urease (URE), (**b**) alkaline phosphatase (ALP), (**c**) sucrase (SUC), and (**d**) catalase (CAT) under different hydrochar treatments. Treatments: CK, no hydrochar addition; D1, 1% hydrochar produced at 160 °C; D2, 2% hydrochar produced at 160 °C; G1, 1% hydrochar produced at 240 °C; and G2, 2% hydrochar produced at 240 °C. Different lowercase letters represent significant differences between different treatments at *p* < 0.05; * represent significant at the 0.05 level (*p* < 0.05); *** represent significant at the 0.001 level (*p* < 0.001).

**Figure 4:**
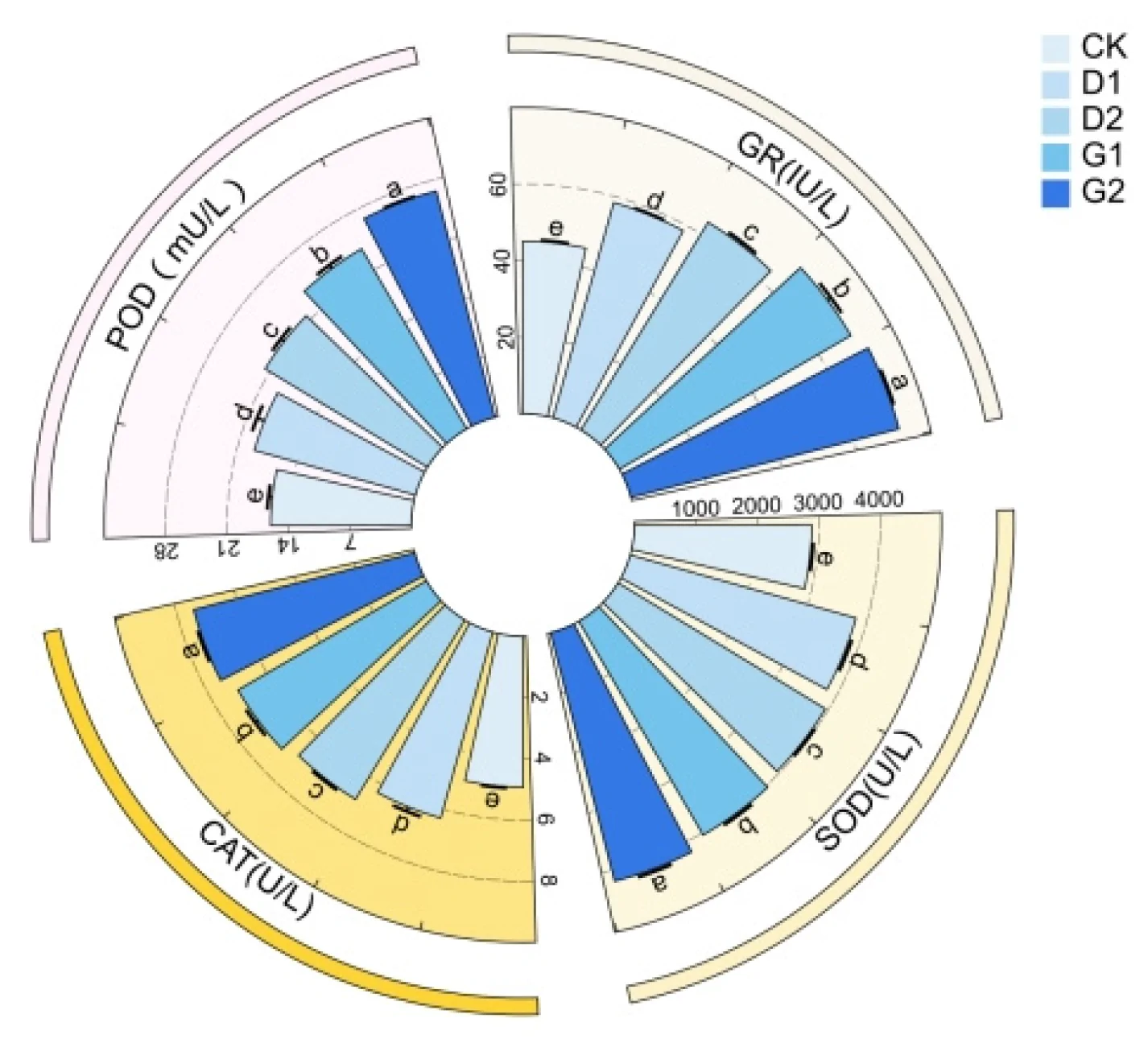
Antioxidant enzyme activities of plants in response to different treatments. Treatments: CK, no hydrochar addition; D1, 1% hydrochar produced at 160 °C; D2, 2% hydrochar produced at 160 °C; G1, 1% hydrochar produced at 240 °C; and G2, 2% hydrochar produced at 240 °C. Different lowercase letters represent significant differences between different treatments at *p* < 0.05.

**Figure 5:**
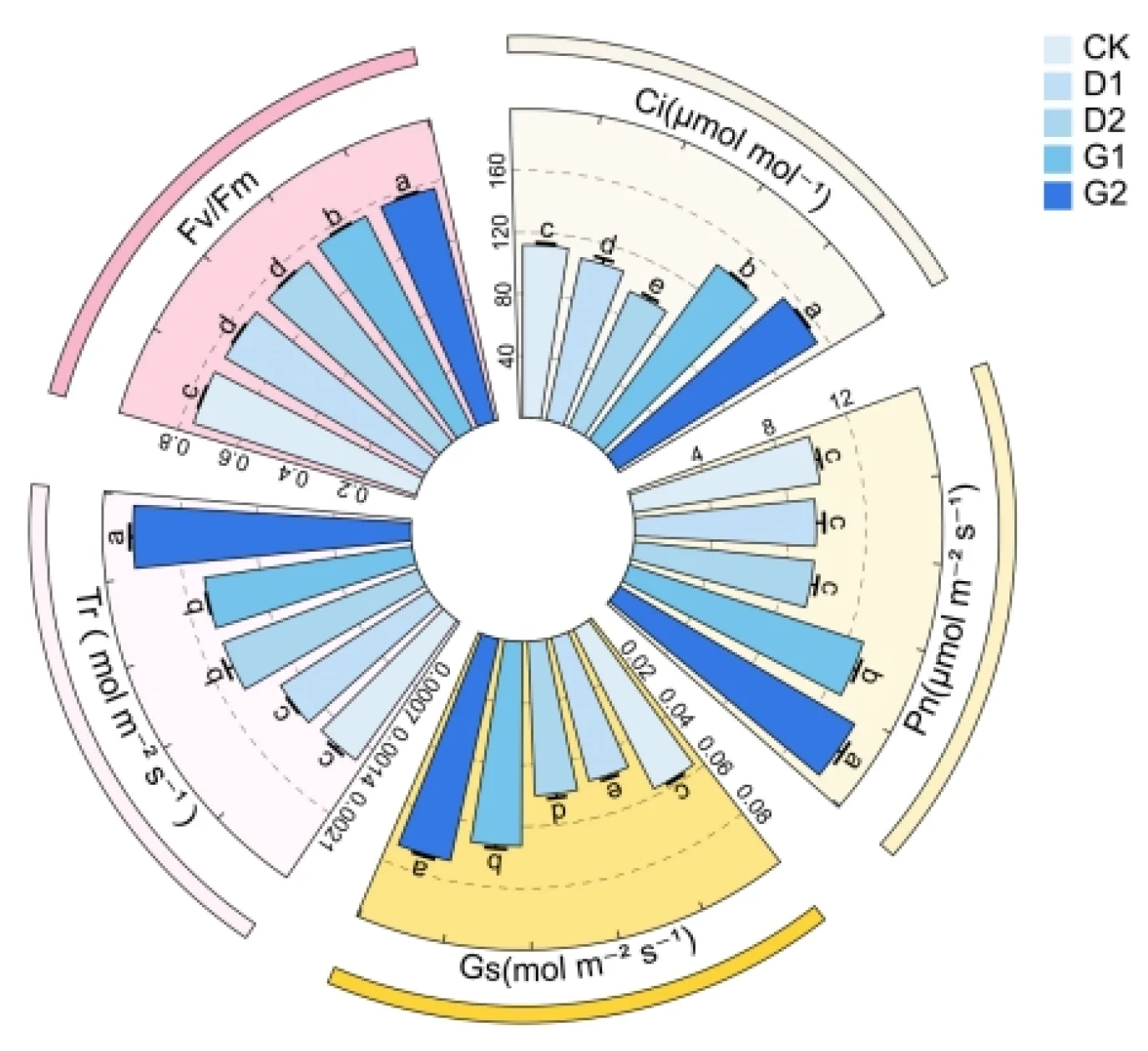
Photosynthetic parameters of plants under different treatments. Treatments: CK, no hydrochar addition; D1, 1% hydrochar produced at 160 °C; D2, 2% hydrochar produced at 160 °C; G1, 1% hydrochar produced at 240 °C; and G2, 2% hydrochar produced at 240 °C. Different lowercase letters represent significant differences between different treatments at *p* < 0.05.

**Figure 6:**
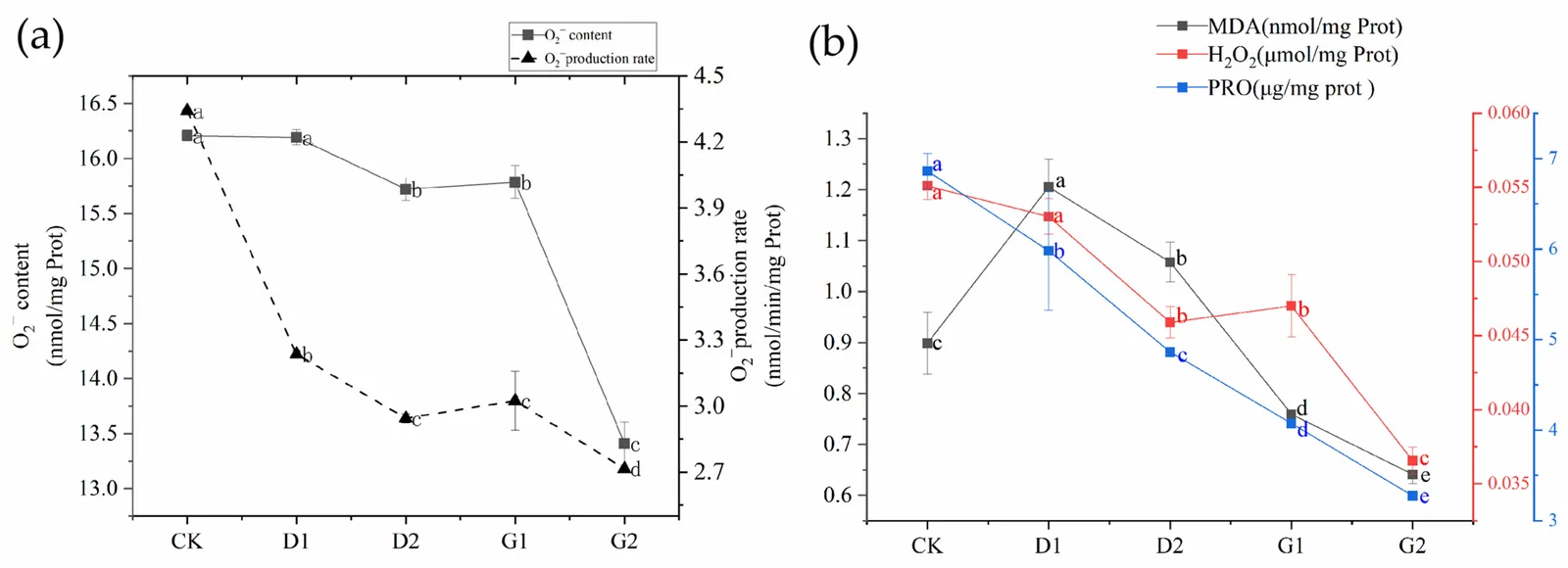
Oxidative stress parameters in foxtail millet under different hydrochar treatments. (**a**) O_2_^−^ content and production rate; (**b**) MDA, H_2_O_2_, and Pro contents. Treatments: CK, no hydrochar addition; D1, 1% hydrochar produced at 160 °C; D2, 2% hydrochar produced at 160 °C; G1, 1% hydrochar produced at 240 °C; and G2, 2% hydrochar produced at 240 °C. Different lowercase letters represent significant differences between different treatments at *p* < 0.05.

**Figure 7:**
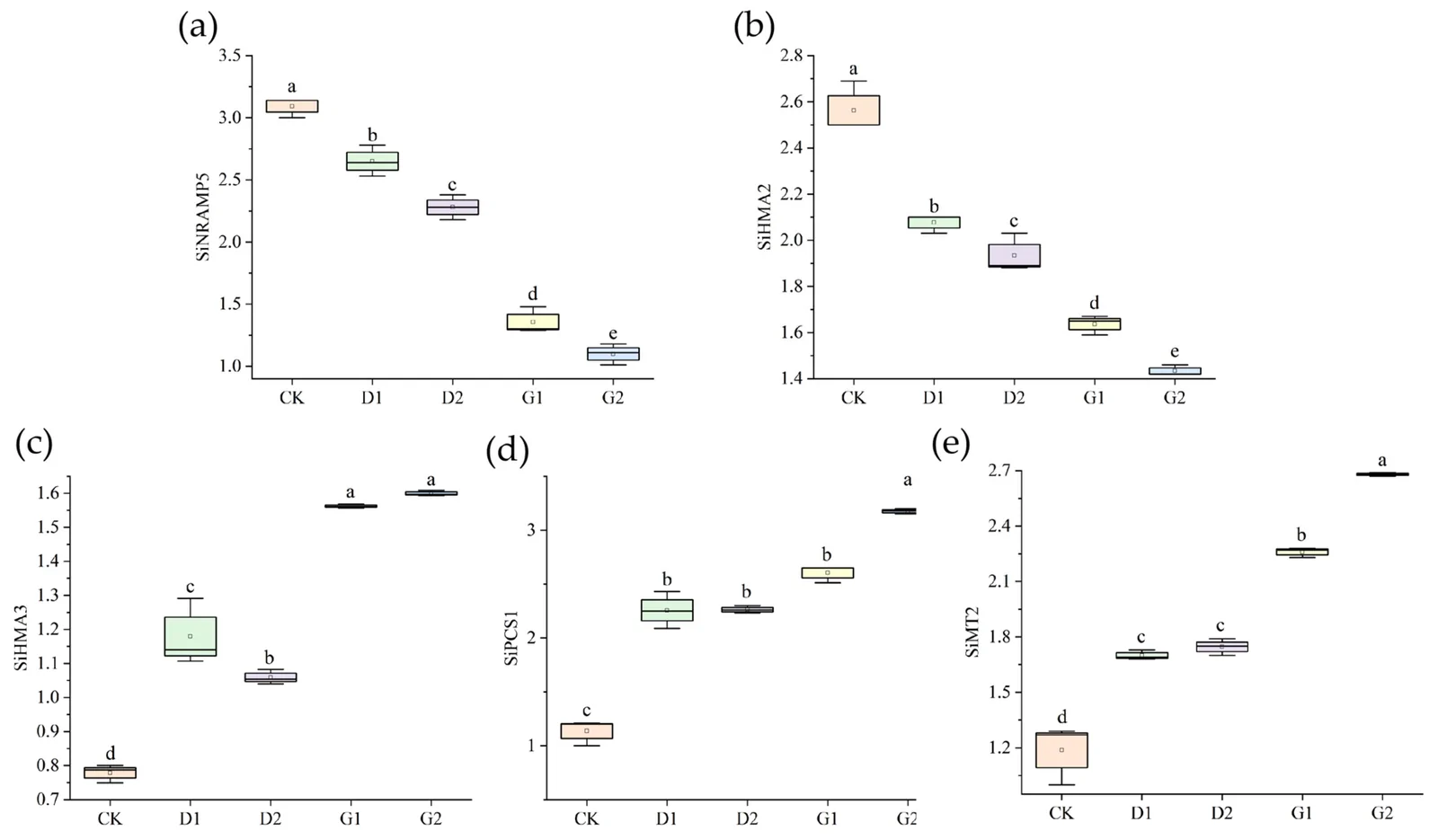
Expression of resistance-related genes (**a**) *SiNRAMP5*, (**b**) *SiHMA2*, (**c**) *SiHMA3*, (**d**) *SiPCS1*, and (**e**) *SiMT2* in foxtail millet under different treatments. Treatments: CK, no hydrochar addition; D1, 1% hydrochar produced at 160 °C; D2, 2% hydrochar produced at 160 °C; G1, 1% hydrochar produced at 240 °C; and G2, 2% hydrochar produced at 240 °C. Different lowercase letters represent significant differences between different treatments at *p* < 0.05.

**Figure 8:**
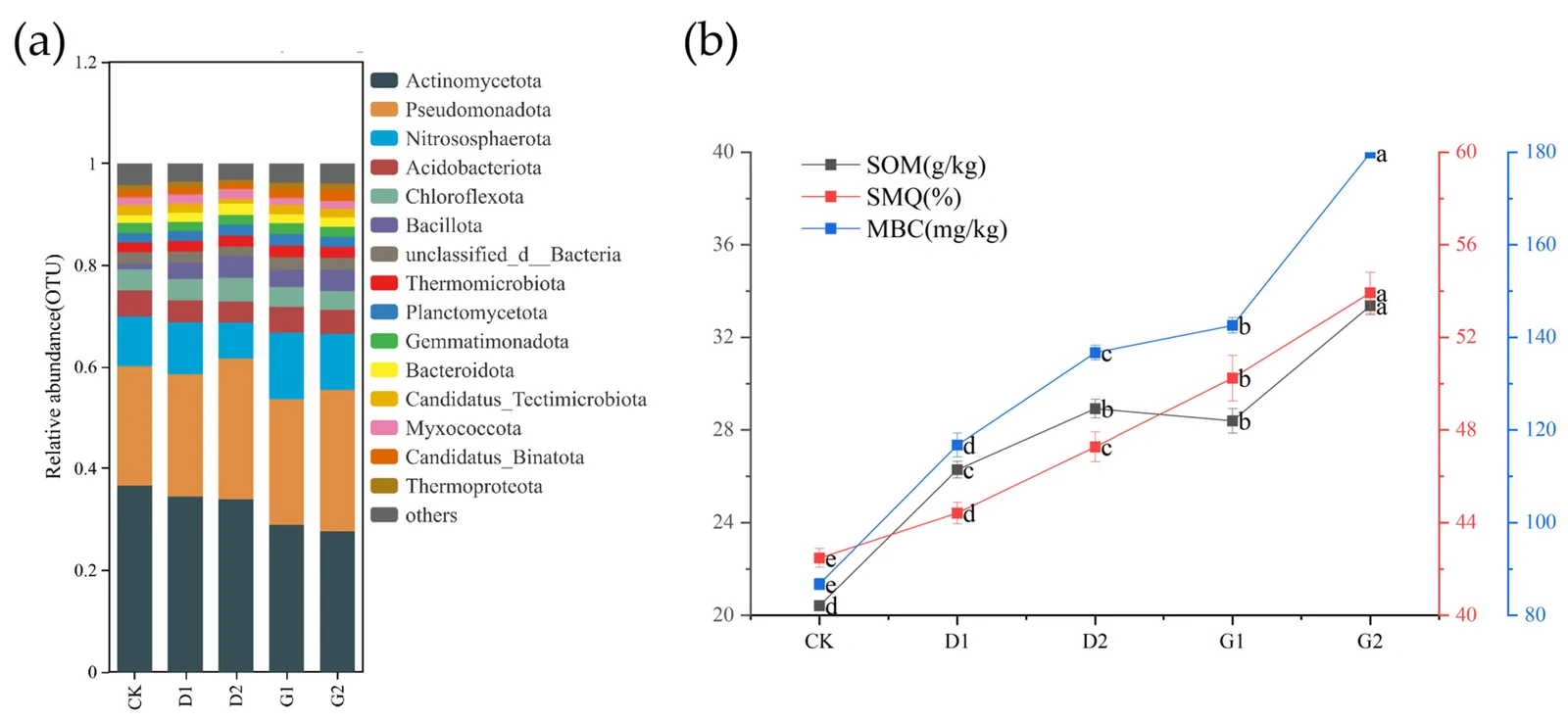
(**a**) Bacterial community composition (phylum level) and (**b**) SOM, MBC, and SMQ under different treatments. Treatments: CK, no hydrochar addition; D1, 1% hydrochar produced at 160 °C; D2, 2% hydrochar produced at 160 °C; G1, 1% hydrochar produced at 240 °C; and G2, 2% hydrochar produced at 240 °C. Different lowercase letters represent significant differences between different treatments at *p* < 0.05.

**Figure 9:**
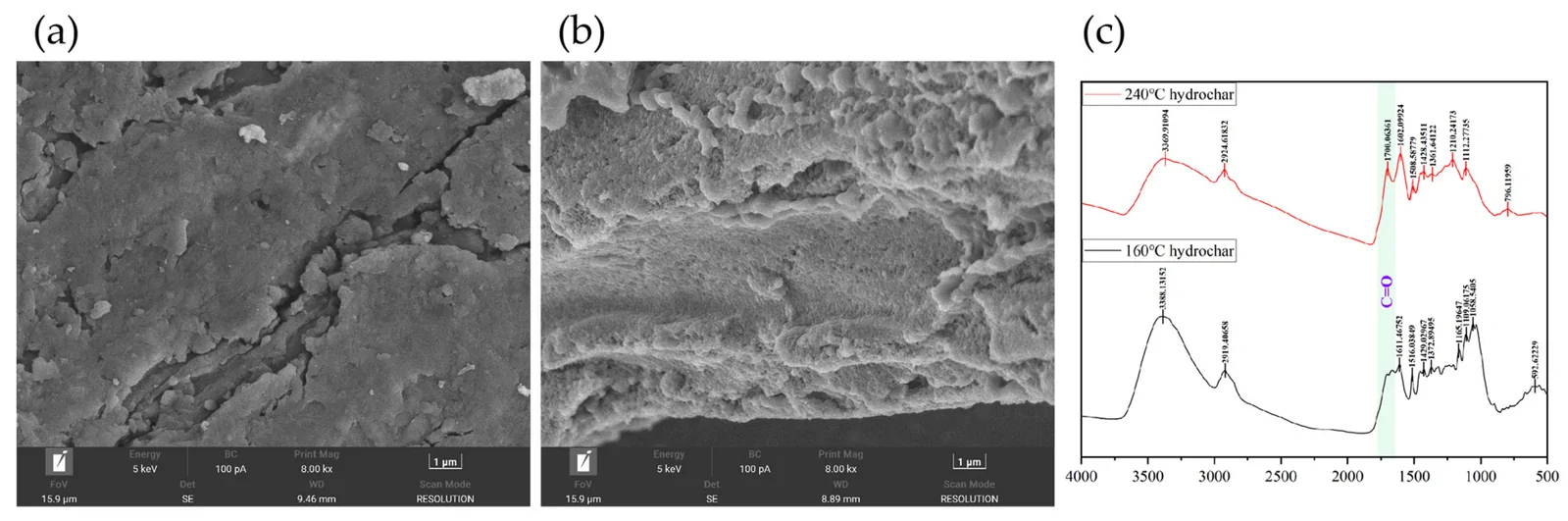
(**a**) SEM image of hydrochar prepared at 160 °C, (**b**) SEM image of hydrochar prepared at 240 °C, and (**c**) FTIR spectra of the hydrochars.

**Figure 10:**
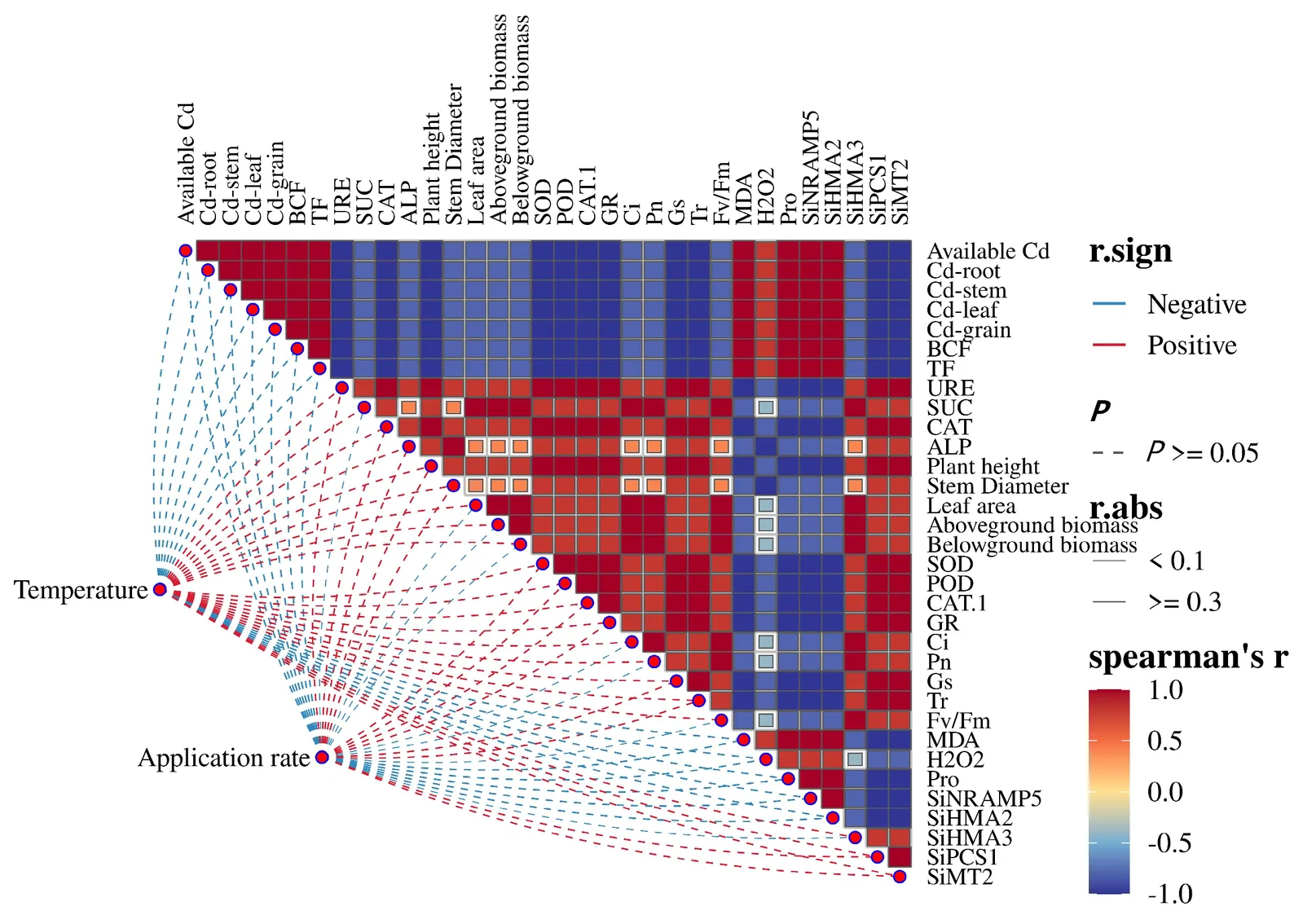
Spearman’s correlation analysis. Network heatmap showing correlations among indicators and between environmental factors and indicators. Spearman’s r, Spearman correlation coefficient; r.sign, positive or negative correlation; r.abs, threshold of absolute Mantel’s r. Squares with white borders indicate nonsignificant Spearman correlations (*p* ≥ 0.05). Dots represent the nodes corresponding to environmental factors and indicators in the correlation network. Dashed lines indicate nonsignificant Mantel correlations (*p* ≥ 0.05).

**Table 1:** Growth characteristics and biomass of foxtail millet.

Treatment	Plant Height (cm)	Stem Diameter (mm)	Leaf Area (cm^2^)	Aboveground Biomass (g)	Belowground Biomass (g)
CK	61.31 ± 2.60 ^c^	2.57 ± 0.36 ^b^	19.18 ± 0.61 ^d^	7.51 ± 0.66 ^c^	0.89 ± 0.14 ^b^
D1	57.11 ± 5.64 ^c^	1.98 ± 0.10 ^c^	25.02 ± 3.93 ^c^	9.71 ± 1.43 ^b^	0.83 ± 0.26 ^b^
D2	60.92 ± 5.01 ^c^	2.83 ± 0.33 ^b^	24.94 ± 1.52 ^c^	8.37 ± 1.54 ^bc^	0.86 ± 0.07 ^b^
G1	67.88 ± 2.67 ^b^	2.64 ± 0.36 ^b^	32.30 ±7.06 ^b^	12.03 ± 0.49 ^a^	1.14 ± 0.11 ^ab^
G2	77.79 ± 7.07 ^a^	3.68 ± 0.47 ^a^	45.94 ± 9.09 ^a^	13.09 ± 1.55 ^a^	1.26 ± 0.09 ^a^

Treatments: CK, no hydrochar addition; D1, 1% hydrochar produced at 160 °C; D2, 2% hydrochar produced at 160 °C; G1, 1% hydrochar produced at 240 °C; and G2, 2% hydrochar produced at 240 °C. Different lowercase letters represent significant differences between different treatments at *p* < 0.05.

**Table 2:** Pore structure parameters of hydrochar.

Hydrochar Type	Micropore Area (m^2^/g)	Mesopore Area (m^2^/g)	Specific Surface Area (m^2^/g)
Hydrochar-160 °C	0.66	0.46	0.83
Hydrochar-240 °C	0.67	2.50	3.11

## Data Availability

The original contributions presented in this study are included in the article/Supplementary Materials. Further inquiries can be directed to the corresponding author. Data are available upon reasonable request.

## Supplementary Materials

The following supporting information can be downloaded at: https://www.mdpi.com/article/10.3390/toxics14100855/s1, Table S1. Effects of hydrochar pyrolysis temperature and application rate on soil, analysed by two-way ANOVA.

## Abbreviations

The following abbreviations are used in this manuscript:

ALP	Alkaline phosphatase
ANOVA	Analysis of variance
ELISA	Enzyme-linked immunosorbent assay
GR	Glutathione reductase
MBC	Microbial biomass carbon
SEM	Scanning electron microscopy
SOM	Soil organic matter
TF	Translocation factor
