# Supplementary material for: Effects of Hydrochar Application on Soil Cadmium Immobilisation and Foxtail Millet Growth
Source: Toxics. 2026 Sep 25;14(10):855. doi: 10.3390/toxics14100855 (PMC13709503; doi:10.3390/toxics14100855)
Supplement: Supplementary file 1 [file toxics-14-00855-s001.zip › toxics-4551700-supplementary.pdf]

Supplementary Material

Effects of Hydrochar Application on Soil Cadmium Immobilisation and Foxtail Millet Growth

Wenchi Guo, Yan Zhang, Jinpeng Yu, Yaping Li, Fei Han, Hong Pan, Hui Wang, Quangang Yang,
Zhongchen Yang, Yanhong Lou * and Yuping Zhuge *

National Engineering Research Centre for Efficient Utilization of Soil and Fertilizer Resources, College of
Resources and Environment, Shandong Agricultural University, Daizong Road, Tai'an City 271018, China

* Correspondence: yanhonglou1985@163.com (Y.L.); zhugeyp@sdau.edu.cn (Y.Z.)

Table S1. Effects of hydrochar pyrolysis temperature and application rate on soil, analysed by two-way ANOVA.

Factors	Soil available Cd	URE	SUC	CAT	ALP
temperature	***	***	***	***	***
Application rate		*	*	***	***
temperature*Application rate			***	***	*

Factors	Cd content in roots	Cd content in stems	Cd content in leaves	Cd content in grains	GR
temperature	***	***	***	***	***
Application rate	***	***	***	***	***
temperature*Application rate	***	***	***		**

Factors	SOD	POD	CAT	MDA	H_2O_2
temperature	***	***	***	***	***
Application rate	***	***	***	***	***
temperature*Application rate					

Factors	PRO	O_2^- content	O_2^- production	Tr	Gs

temperature	***	***	***	***	***
Application rate	**	***	***	***	***
temperature*Application rate		***		***	**

Factors	Pn	Ci	Fv/Fm	SiNRAMP5	SiHMA2
temperature	***	***	***	***	***
Application rate	**			***	***
temperature*Application rate	**	***	*		

Factors	SiHMA3	SiPCS1	SiMT2	MBC	SOM
temperature	***	***	***	***	***
Application rate		***	***	***	***
temperature*Application rate	**	***	*		

Significance levels are denoted as follows: * significant at the 0.05 level ($p < 0.05$); ** significant at the 0.01 level ($p < 0.01$); *** significant at the 0.001 level ($p < 0.001$).
